# *X*-to-autosome expression and *msl-2* transcript abundance correlate among *Drosophila melanogaster* somatic tissues

**DOI:** 10.7717/peerj.771

**Published:** 2015-02-17

**Authors:** Steven P. Vensko II, Eric A. Stone

**Affiliations:** 1 Program in Genetics, North Carolina State University, Raleigh, NC, USA; 2 Department of Biological Sciences, North Carolina State University, Raleigh, NC, USA

**Keywords:** H4K16Ac, Male-Specific Lethal complex, *Drosophila melanogaster*, Dosage compensation complex, Dosage compensation

## Abstract

In *Drosophila melanogaster*, the male-specific lethal (MSL) complex has been studied extensively for its role in upregulating male *X*-linked genes. Recent advances in high-throughput technologies have improved our understanding of how the MSL complex mediates dosage compensation through chromosome-wide chromatin modifications. Most studies, however, have focused on cell line models that cannot reflect any potential heterogeneity of *in vivo* dosage compensation. Comparisons between cell line and organismal gene-level dosage compensation upregulation suggest the possibility of variation in MSL complex activity among somatic tissues. We hypothesize the degree, up to but not exceeding 2-fold, to which the MSL complex upregulates male *X*-linked genes varies quantitatively by tissue type. In this model, MSL complex abundance acts as a rheostat to control the extent of upregulation. Using publicly available expression data, we provide evidence for our model in *Drosophila* somatic tissues. Specifically, we find *X*-to-autosome expression correlates with the tissue-specific expression of *msl-2* which encodes an essential male-specific component of the MSL complex. This result suggests MSL complex mediated dosage compensation varies quantitatively by tissue type. Furthermore, this result has consequences for models explaining the organismal-scale molecular and evolutionary consequences of MSL-mediated dosage compensation.

## Introduction

Dosage compensation, the mechanism believed to offset sex differences in transcript abundance due to sex chromosome aneuploidy, is found across many genera in unique forms (Prestel, Feller & Becker, 2010). In *Drosophila melanogaster*, dosage compensation is mediated by the male-specific lethal (MSL) complex, which upregulates gene expression on the *X* chromosome by approximately two-fold (Straub et al., 2005). The MSL complex, reviewed by Conrad & Akhtar (2012), consists of at least five proteins (*Male-Specific Lethal-1 (MSL-1)*, *Male-Specific Lethal-2 (MSL-2)*, *Male-Specific Lethal-3 (MSL-3)*, *Maleless (MLE)*, and *Males Absent on the First (MOF)*) as well as two redundant non-coding RNAs (*RNA on the X 1 (roX1)* and *RNA on the X 2 (roX2*)). The MSL complex binds to the male *X* chromosome, resulting in acetyaltion of lysine 16 of the fourth histone core (H4K16Ac), and consequently preventing formation of 30 nm chromatin fibers (Shogren-Knaak et al., 2006; Robinson et al., 2008). This induces “looser” chromatin and is believed to increase accessibility for DNA-binding proteins (Bell et al., 2010). Although the mechanism underlying MSL complex-dependent upregulation remains contested, both increased transcriptional elongation and increased RNA polymerase II (PolII) promoter density have been proposed (Larschan et al., 2011; Conrad et al., 2012; Ferrari et al., 2013; Straub & Becker, 2013).

Our understanding of *Drosophila* MSL complex-dependent dosage compensation (hereafter referred to as dosage compensation or DC) has advanced with the development of high-throughput assays over the past decade. These studies contribute insight into many aspects of dosage compensation, including the signals identifying the male *X* chromosome as the primary target of the MSL complex (Alekseyenko et al., 2006; Alekseyenko et al., 2008; Straub et al., 2008; Alekseyenko et al., 2012) and the extent to which the MSL complex upregulates male *X*-linked genes (Straub et al., 2005; Deng & Meller, 2006; Hamada et al., 2005). S2 cells have been commonly utilized for these studies due to their ease of genetic manipulation and MSL complex activity (Straub et al., 2008; Hamada et al., 2005; Alekseyenko et al., 2006; Alekseyenko et al., 2012; Straub et al., 2005). While S2 cells have been fundamental toward understanding the molecular mechanism of DC, they do not reflect heterogeneity potentially present at an organismal level. Most genes show variable expression patterns across tissues; thus, if dosage compensation shows differential activity across tissues then a cell-line model of dosage compensation cannot faithfully replicate the complex patterns of DC within whole organisms. For this reason, an appreciation of organismal-level DC complexity is essential for models attempting to clarify the global molecular and evolutionary consequences of dosage compensation on *X*-linked genes (Bachtrog, Toda & Lockton, 2010; Mikhaylova & Nurminsky, 2011). For example, work by Mikhaylova & Nurminsky (2011) suggests that tissue specificity may play a role in the migration of genes off of the *Drosophila X* chromosome to avoid deleterious overexpression by dosage compensation. If the degree of dosage compensation varies by tissue, quantifying tissue-level heterogeneity would help clarify the relationship between DC and expression tissue specificity. This may, in turn, lead to a better understanding of the selective pressures that may have influenced *Drosophila X*-to-autosome retrotranspositions.

We propose that somatic *Drosophila* tissues, unlike homogeneous S2 cells, show significant variation in dosage compensation activity. This suggests the action of dosage compensation on any *X*-linked gene is conditional upon the extent of MSL complex activity across each tissue in which it is expressed. Ours is not a radical proposal: strong evidence against MSL complex-mediated dosage compensation in the testis already exists (Bachiller & Sánchez, 1986; Rastelli & Kuroda, 1998; Meiklejohn et al., 2011). We simply advocate for an analog perspective on dosage compensation, rather than a digital one in which the complex is either present and active or not. Our hypothesis is largely motivated by observations by Deng & Meller (2006) showing that dosage compensated *X*-linked genes tend to be upregulated in both S2 cells and third instar larval males but at drastically different levels. These differences in upregulation suggest dosage compensation patterns observed within cell lines do not translate to an organismal context, possibly due to variation in dosage compensation activity among third instar larval male somatic tissues. Furthermore, recent work by Nozawa et al. (2014) provides support for this hypothesis using interspecific chromosome-level expression comparisons to show dosage compensation on the *D. psuedoobscura* neo-*X* chromosome varies between male heads, gonads, and male carcasses. Here we present an intraspecific approach providing evidence for extensive variation in dosage compensation among a variety of *Drosophila melanogaster* somatic tissues.

## Methods

FlyAtlas data were collected from Gene Expression Omnibus (GEO) series entry GSE7763 (last updated May 29, 2013) (Chintapalli, Wang & Dow, 2007). Tissues were filtered to include only non-female-specific tissues. Whole organism “tissue” entries were also removed. A complete listing of tissues can be found in Table S1. Probe sets were mapped to FBgn IDs using the FB2014_03 Release *Drosophila melanogaster* exon summary file (Marygold et al., 2013). In cases where FBgn IDs had multiple representative probe sets, the mean expression intensity was calculated. Genes were filtered for expression on a tissue-by-tissue basis. For each tissue, genes were classified as expressed if they had “present” calls for all four FlyAtlas replicates and had *log*_2_ intensity values ≥6 for all four FlyAtlas replicates (Stenberg et al., 2009; Philip, Pettersson & Stenberg, 2012). Only expressed genes were utilized to estimate X and autosomal expression for each tissue.

Genes were classified as *X*-linked or autosomal based on the FB2014_03 Release gene map table from FlyBase (Marygold et al., 2013). Sex-bias classifications were acquired from the Sex Bias Database (SEBIDA v3.1) (Gnad & Parsch, 2006). In particular, we used the “meta” sex-bias classifications which utilize expression data from a variety of sources (Ranz et al., 2003; Parisi et al., 2004; Ayroles et al., 2009). Genes were classified as non-sex-biased if classified as “unbiased” by SEBIDA and were classified as sex-biased otherwise.

Custom R scripts (available upon request) were created for statistical analysis (R Core Team, 2013). All *X*-to-autosome expression ANOVAs used mean expression measurements from *X*-linked and autosomal genes (such that the mean expression tissue-level replicate estimate is the mean expression of all genes expressed within that tissue and that replicate). Correlations were performed using the mean of replicate *X*-to-autosome mean expression values and the mean of replicate *msl-2* expression measurements. All figures were created using the ggplot2 R library (Wickham, 2009). Tissue-level *X*-to-autosome and MSL complex components replicate expression estimates can be found in Table S1 and tissue-level *X*-to-autosome and MSL complex components mean expression estimates can be found in Table S2.

## Results and Discussion

### Variation in male *Drosophila X* chromosome upregulation across somatic tissues

**Figure 1:**
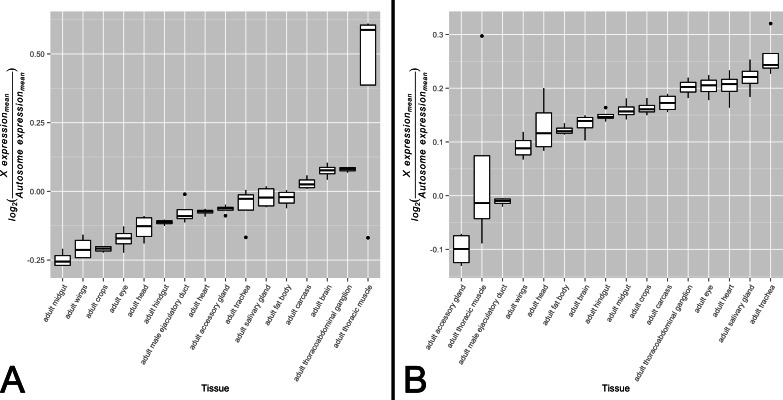
*X*-to-autosome expression variation among somatic tissues. *X*-to-autosome expression estimates were calculated for all four FlyAtlas replicates for each tissue using the log_2_ transformed ratios of the mean expression of *X*-linked expressed genes to mean expression of autosomal expressed genes for both the (A) non-sex-biased gene set and (B) sex-biased gene set. Tissues are sorted by their median log_2_ ratio among the FlyAtlas replicates.

There is ample evidence that the expression patterns of X-linked genes vary in their tissue distribution (Chintapalli, Wang & Dow, 2007). For these genes to exhibit varying levels of dosage compensation on an organismal level, dosage compensation must also be differentially active across the male somatic tissues in which these genes are expressed. Noting that the primary role of the MSL complex in dosage compensation is to offset male monosomy by upregulating a majority of *X*-linked genes, it stands to reason that decreased complex activity should associate with a larger difference in expression levels between the *X* and the autosomes. If the degree of difference varies quantitatively across tissues, this would provide evidence consistent with an analog model of tissue-specific dosage compensation. While this approach appears straight-forward, one must be cognizant of the differing evolutionary histories of the *X* chromosome and autosomes that may dilute any signal of *X* chromosome dosage compensation upregulation. Taking this into consideration, we partitioned genes into a non-sex-biased set and a sex-biased set. We expect the non-sex-biased gene set to be dosage compensated, show similar enrichment on the *X* chromosome and autosomes, and be less likely to be regulated sex-specific mechanisms. We expect the sex-biased gene set, on the other hand, to be likely experiencing some level of dosage compensation but for this signal to be diluted by sex-specific regulatory mechanisms and an unequal distribution between the *X* chromosome and autosomes. These two gene sets, therefore, provide us the ability to detect any signal of dosage compensation in the non-sex-biased gene set while ensuring sex-specific mechanisms and differing gene content between the *X* chromosome and autosomes are not driving the signal. We tested for variation in *X*-to-autosome expression among tissues for both gene sets by calculating the difference between mean *X* chromosome gene expression and mean autosome gene expression. We found significant variation in *X*-to-autosome expression among somatic adult tissues for both the non-biased gene set (Fig. 1A, *F*_15,48_ ≈ 9.40, *p* < 0.005) and sex-biased gene set (Fig. 1B, *F*_15,48_ ≈ 13.64, *p* < 0.005). These trends were also observed among non-sex-biased genes within larval tissues (Fig. S1, *F*_7,24_ ≈ 279.52, *p* < 0.005). In line with our expectations, the adult testis shows the lowest *X*-to-autosome expression among adult tissues (see Fig. S2). Interestingly, there is no significant correlation between the non-sex-biased gene set and sex-biased gene set which suggests they are under differing transcriptional regulatory regimes. While these results hint toward the possibility of variation in dosage compensation activity among somatic tissues, other mechanisms may instead be responsible. We sought further evidence of our hypothesis by interrogating tissue-specific variation of the MSL complex itself.

### Variation in MSL complex abundance across somatic tissues

**Figure 2:**
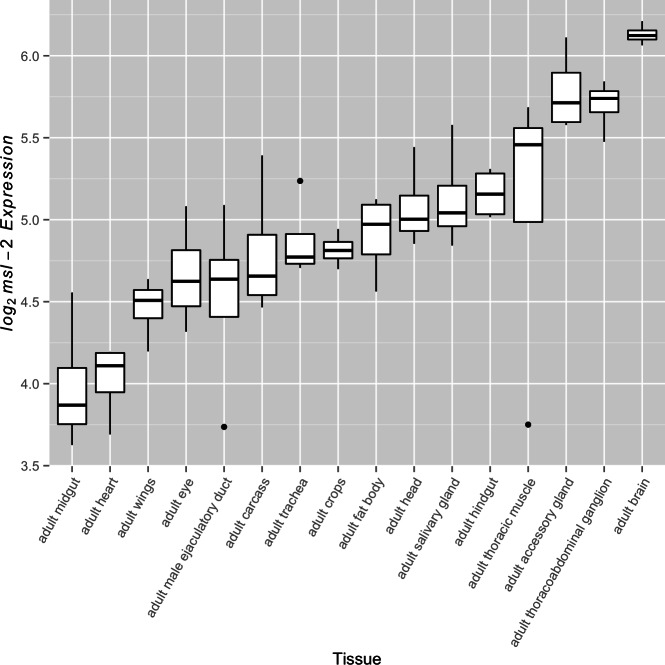
*msl-2* expression variation among somatic tissues. *msl-2* expression estimates were retrieved for all four FlyAtlas replicates for each tissue. Tissues are sorted by their median log_2_ *msl-2* intensity among the FlyAtlas replicates.

For the MSL complex to be active, it must first be present. Previous studies have shown that after generating the MSL complex in females through introduction of a functionally spliced *msl-2* transgene, a quantitative increase in MSL complex abundance leads to increased binding to the female *X* chromosome. A common strategy in these studies is to modulate transcript levels, specifically those of *msl-2*, which encodes a subunit essential for proper MSL complex formation (Demakova et al., 2003; Dahlsveen et al., 2006; Kelley et al., 1995; Oh, Park & Kuroda, 2003; Park et al., 2002; Hamada et al., 2005). These studies commonly utilize females to study the relationship between MSL complex abundance and dosage compensation through upregulation of the naturally absent MSL-2 protein; however, they establish a relationship that is expected to be relevant in males. While both *msl-1* and *msl-2* serve fundamental roles in formation of the MSL complex, *msl-1* transcript abundance exceeds *msl-2* transcript abundance in every FlyAtlas adult tissue suggesting MSL-2 may be the limiting factor. This led us to examine variation in *msl-2* transcript abundance levels across tissues as a surrogate for differential complex activity. We found significant variation for *msl-2* expression among adult somatic tissues (Fig. 2, *F*_15,48_ ≈ 10.44, *p* < 0.005). Once again, this trend was also observed in larval tissues (Fig. S3, *F*_7,24_ ≈ 33.31, *p* < 0.005). Consistent with expectations (Bachiller & Sánchez, 1986; Rastelli & Kuroda, 1998; Meiklejohn et al., 2011), when included in the analysis, the adult testis show the lowest amount of *msl-2* expression (see Fig. S4). It is worth noting other components of the MSL complex also showed significant variation in transcript abundance among somatic adult tissues (*F*_15,48_ ≈ 18.71, *p* < 0.005 for *msl-1*, *F*_15,48_ ≈ 6.17, *p* < 0.005 for *msl-3*, *F*_15,48_ ≈ 37.58, *p* < 0.005 for *mle*, and *F*_15,48_ ≈ 3.49, *p* < 0.005 for *mof*).

### Covariation between *msl-2* transcript abundance and *X*-to-autosome expression across somatic tissues

**Figure 3:**
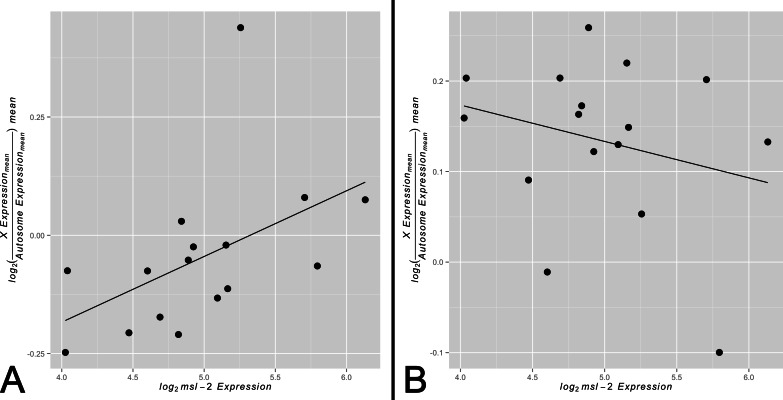
Correlation between *X*-to-autosome expression and *msl-2* expression among somatic tissues. *X*-to-autosome mean expression plotted against its corresponding *msl-2* mean expression for each somatic adult tissue for the (A) non-sex-biased gene set and (B) sex-biased gene set.

By comparing *X* and autosomal expression levels across tissues, we found evidence consistent with tissue-specific variability in *X* chromosome expression relative to autosomal expression. Similarly, we found that the abundance of *msl-2* varies by tissue, suggesting the MSL complex is likely differentially present and active. We sought to test for a correlation between *X*-to-autosome expression and *msl-2* transcript abundance among somatic tissues for both the non-sex-biased and sex-biased gene sets. A scenario in which non-sex-biased genes show a significantly positive correlation between *msl-2* transcript abundance and *X*-to-autosome expression while sex-biased genes show no significant correlation would support our model of varying levels of dosage compensation among adult somatic tissues. Any other outcome would not provide evidence for our model. In support of our hypothesis, we found a significant positive correlation between *msl-2* expression and *X*-to-autosome expression among somatic adult tissues for non-sex-biased gene set (Fig. 3A, *ρ* ≈ 0.68, *p* < 0.005). The adult thoracic muscle is an extreme outlier relative to the other tested tissues suggesting further investigation may yield interesting results. Larval tissues showed a positive but non-significant correlation likely due to its small sample size (Fig. S5, *ρ* ≈ 0.21, *p* > 0.05). The sex-biased gene set shows no significant correlation between *msl-2* expression and *X*-to-autosome expression among somatic adult tissues (Fig. 3B, *ρ* ≈ −0.19, *p* > 0.05). It is worth noting the *msl-2* locus is not *X*-linked and thus *msl-2* expression is not confounded with *X* chromosome expression. Other MSL complex components, when tested individually, do not show any significant correlation between their expression and *X*-to-autosome expression among tissues. While only finding significant effects only for *msl-2* may not seem intuitive, considering *msl-2*’s likely MSL complex-specific role and its required presence for complex formation, it may be the only component with detectable effects.

### Implications and considerations

High-throughput studies have improved our understanding of *Drosophila* dosage compensation; however, substantial questions remain. Here we have contributed evidence for an analog model of dosage compensation in which the degree of transcriptional upregulation activity is tissue specific. This suggests dosage compensation, while bound between 1-fold and 2-fold upregulation, is the product of a gene’s tissue distribution, transcriptional activity *and* abundance of the MSL complex in tissues in which that gene is expressed.

While we anticipate our results will motivate further investigation into the heterogeneity underlying organismal dosage compensation, there are several considerations in the interpretation of our results. Due to the mixed-sex experimental design of the FlyAtlas data, both tissue-specific *msl-2* expression measurements and chromosome-level expression measurements were sampled from a set of equal count of male and female individuals. This is likely diluting the signal of dosage compensation, in which case we may be underestimating both *msl-2* expression variation and *X*-to-autosome expression variation among somatic adult tissues, as well as the strength of the correlation between them. Specifically, although *msl-2* is transcribed in both males and females, its lower abundance in females may be biasing tissue-level expression measurements (Zhou et al., 1995). Likewise, tissue-level *X*-to-autosome expression measurements may be affected by the contribution of female diploid, non-dosage compensated *X* chromosomes to the transcript pool. Nevertheless, we do not believe that signal dilution from the use of mixed-sex data would generate positively misleading results. For a given tissue, one would expect *X*-to-autosome expression to increase with an increasing contribution of female mRNA, due to their diploid *X* chromosomes. Conversely, because *msl-2* is more lowly expressed in females than in males, relative *msl-2* abundance should decrease as the proportion of female mRNA to the mixed-sex expression pool grows. Thus, the inclusion of female mRNA would drive a negative correlation between *msl-2* expression and *X*-to-autosome expression, as opposed to the significantly positive correlation that we report.

Another consideration is the sole reliance of *msl-2* expression as a marker of MSL complex abundance while using FlyAtlas data. The MSL complex consists of at least seven components that are essential for proper function (Conrad & Akhtar, 2012). That said, modulation of *msl-2* expression has been used repeatedly in the literature as a means to vary MSL complex abundance due its fundamental role in complex formation (Demakova et al., 2003; Dahlsveen et al., 2006; Kelley et al., 1995; Oh, Park & Kuroda, 2003; Park et al., 2002; Hamada et al., 2005). In addition, in every FlyAtlas adult tissue, *msl-2* transcript abundance is lower than that of another potentially limiting component, *msl-1*. Taking these relationships into account, we argue *msl-2* expression is a faithful surrogate for MSL complex abundance. It is worth noting that other subunits (*msl-1, msl-3, mle and mof*) do not show any significant correlation between their transcript abundance and *X*-to-autosome expression among somatic adult tissues. This result is not unexpected for some subunits, such as *mof*, that have known roles beyond the MSL complex (Kind et al., 2008). The lack of significant correlations for the remaining subunits support speculation by Conrad & Akhtar (2012) for many of them having roles beyond dosage compensation.

A final consideration is recent evidence of other non-MSL complex-mediated forms of dosage compensation and its implications for understanding organismal level dosage compensation (Philip & Stenberg, 2013). While no formal mechanism has been proposed, work by Philip & Stenberg (2013) suggests some *X*-linked genes are upregulated independently of MSL complex dosage compensation. Nevertheless, further data regarding any interaction between MSL complex-mediated dosage compensation and non-MSL complex-mediated dosage compensation is required for a full understanding of organismal level of dosage compensation.

*Drosophila* dosage compensation, while well-studied, remains a perplexing mechanism in several regards. Here we present evidence of varying levels of dosage compensation activity among somatic adult tissues. This hypothesis, motivated by differences in *X*-linked upregulation between S2 cells and third instar larval males (Deng & Meller, 2006), suggests care must be taken when quantifying the degree to which an *X*-linked gene is upregulated by dosage compensation in *Drosophila* males. Additional data coupled with an improved understanding of the mechanism underlying dosage compensation will be required to conclusively link variation in MSL complex activity among somatic tissues to variation in expression upregulation by the MSL complex in whole organisms. We hope our results motivate further research into better understanding the behavior of dosage compensation within an organismal context.

## Supplemental Information

10.7717/peerj.771/supp-1 Figure S1 Significant variation in *X*-to-autosome expression among *Drosophila* larval somatic tissues Larval somatic tissues show significant variation for *X*-to-autosome expression (*F*_7,24_ ≈ 279.52, *p* < 0.005).

10.7717/peerj.771/supp-2 Figure S2 Significant variation in *X*-to-autosome expression among *Drosophila*tissues (including adult testis) Adult tissues show significant variation for *X*-to-autosome expression (*F*_16,51_ ≈ 11.77, *p* < 0.005). The adult testis shows the greatest difference in *X*-to-autosome expression which follows our expectation.

10.7717/peerj.771/supp-3 Figure S3 Significant variation in *msl-2* expression among *Drosophila* larval somatic tissues Larval somatic tissues show significant variation for *msl-2* expression (*F*_7,24_ ≈ 33.31, *p* < 0.005).

10.7717/peerj.771/supp-4 Figure S4 Significant variation in *msl-2* expression among *Drosophila* adult tissues (including adult testis) Adult tissues show significant variation for *X*-to-autosome expression (*F*_16,51_ ≈ 20.17, *p* < 0.005). The adult testis expectedly shows the lowest level of msl-2 expression.

10.7717/peerj.771/supp-5 Figure S5 Positive correlation between *X*-to-autosome expression and *msl-2* expression among larval tissues While we did not find a significant correlation, we observed a slight positive correlation between *X*-to-autosome expression and *msl-2* among non-sex-biased genes in larval tissues (*ρ* ≈ 0.21, *p* > 0.05).

10.7717/peerj.771/supp-6 Table S1 Tissue-level X-to-autosome and MSL complex components replicate expression estimates

10.7717/peerj.771/supp-7 Table S2 Tissue-level X-to-autosome values and MSL complex components mean expression estimates
