# Supplementary figures and images for: X-to-autosome expression and msl-2 transcript abundance correlate among Drosophila melanogaster somatic tissues
Source: PeerJ. 2015 Feb 17;3:e771. doi: 10.7717/peerj.771 (PMC4338770; doi:10.7717/peerj.771)

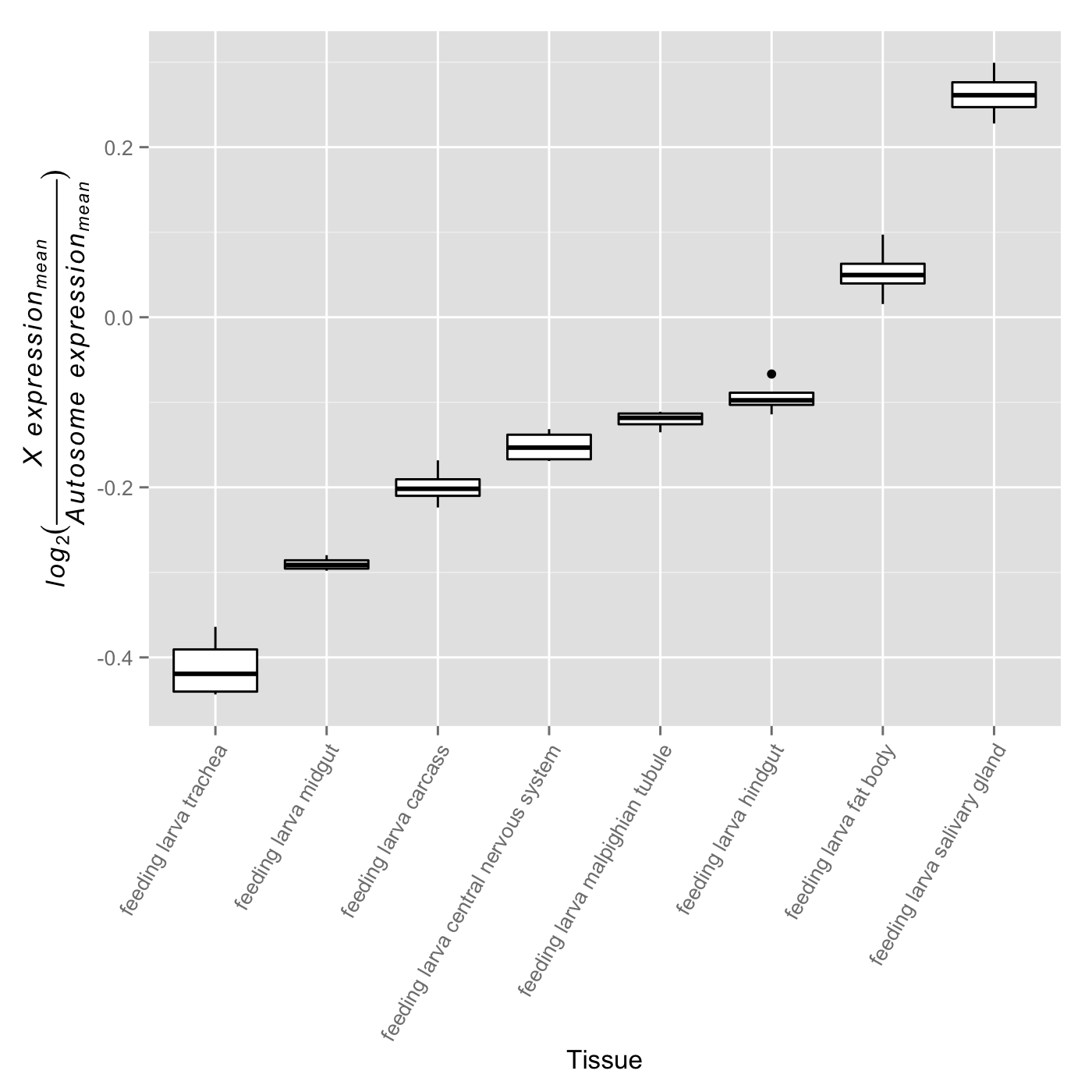

Supplement: Figure S1 — Larval somatic tissues show significant variation for X-to-autosome expression (F7,24 ≈ 279.52, p < 0.005). [file peerj-03-771-s001.png]

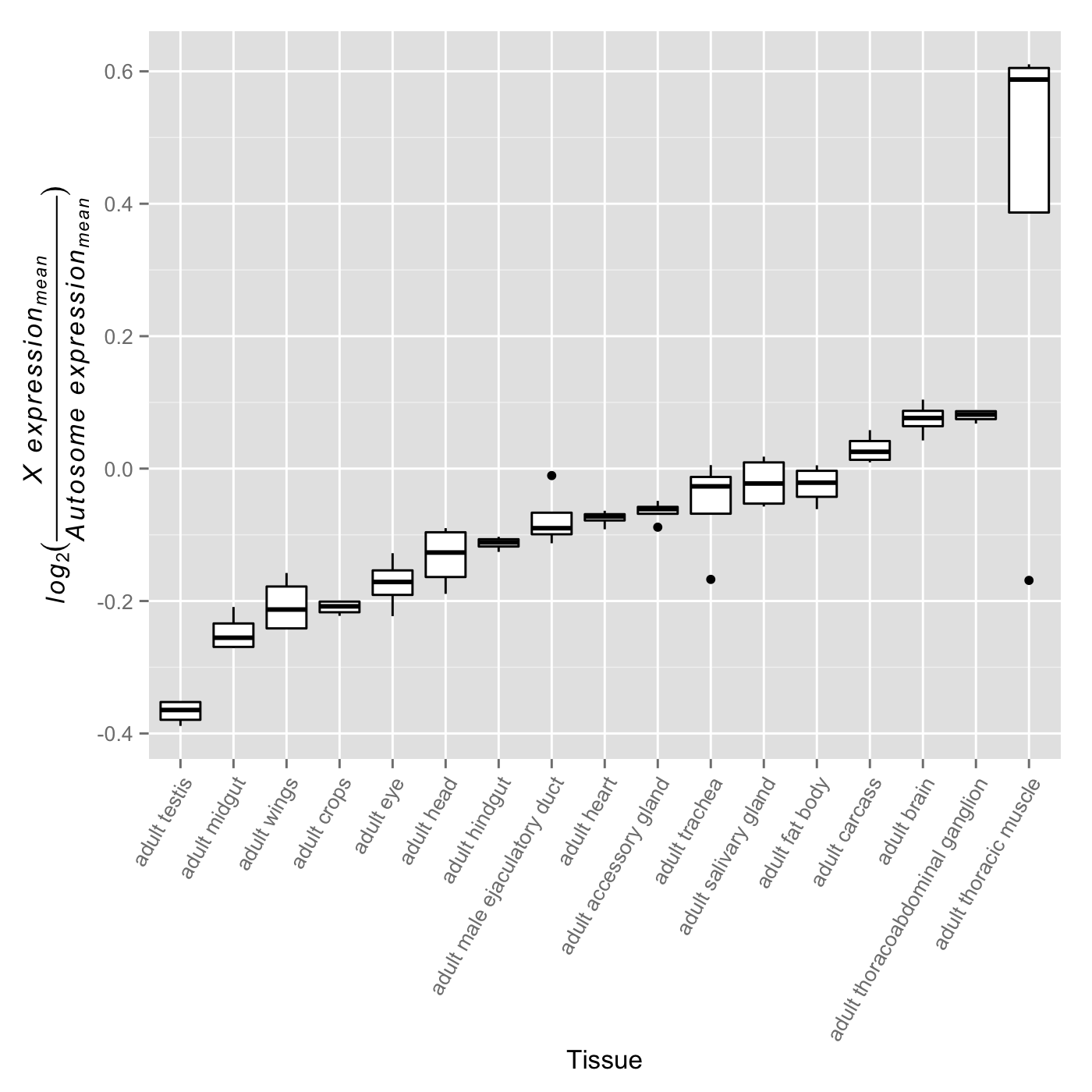

Supplement: Figure S2 — Adult tissues show significant variation for X-to-autosome expression (F16,51 ≈ 11.77, p < 0.005). The adult testis shows the greatest difference in X-to-autosome expression which follows our expectation. [file peerj-03-771-s002.png]

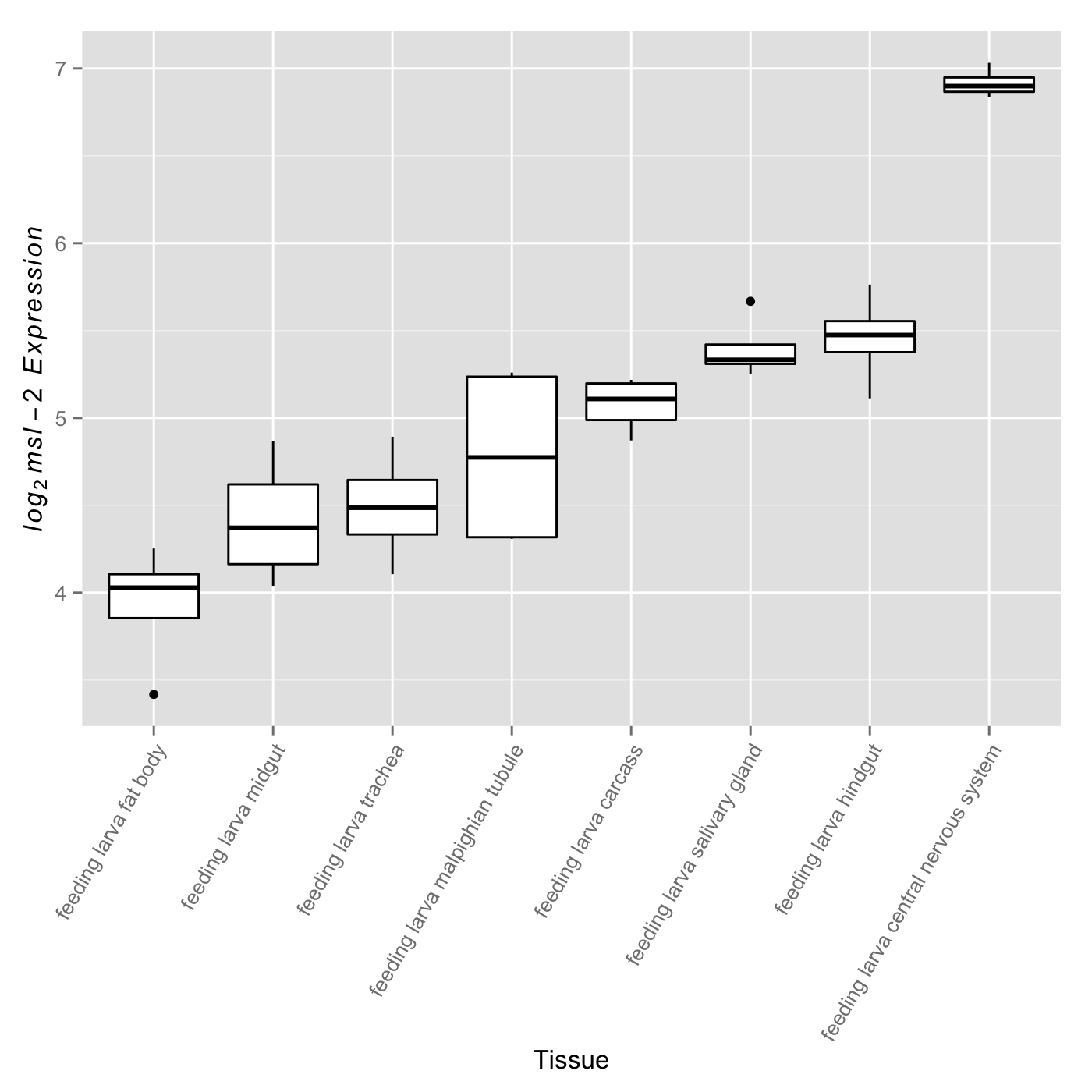

Supplement: Figure S3 — Larval somatic tissues show significant variation for msl-2 expression (F7,24 ≈ 33.31, p < 0.005). [file peerj-03-771-s003.png]

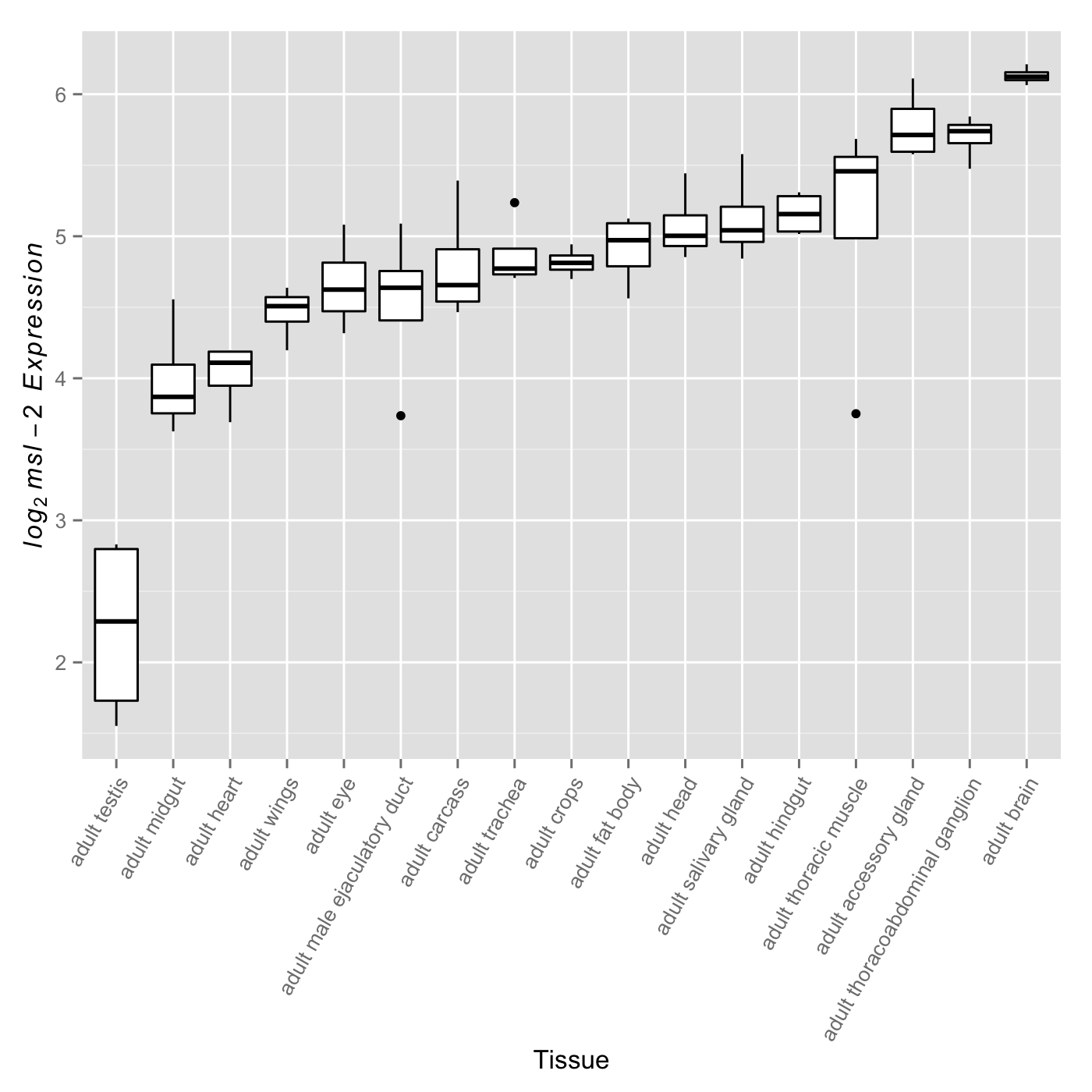

Supplement: Figure S4 — Adult tissues show significant variation for X-to-autosome expression (F16,51 ≈ 20.17, p < 0.005). The adult testis expectedly shows the lowest level of msl-2 expression. [file peerj-03-771-s004.png]

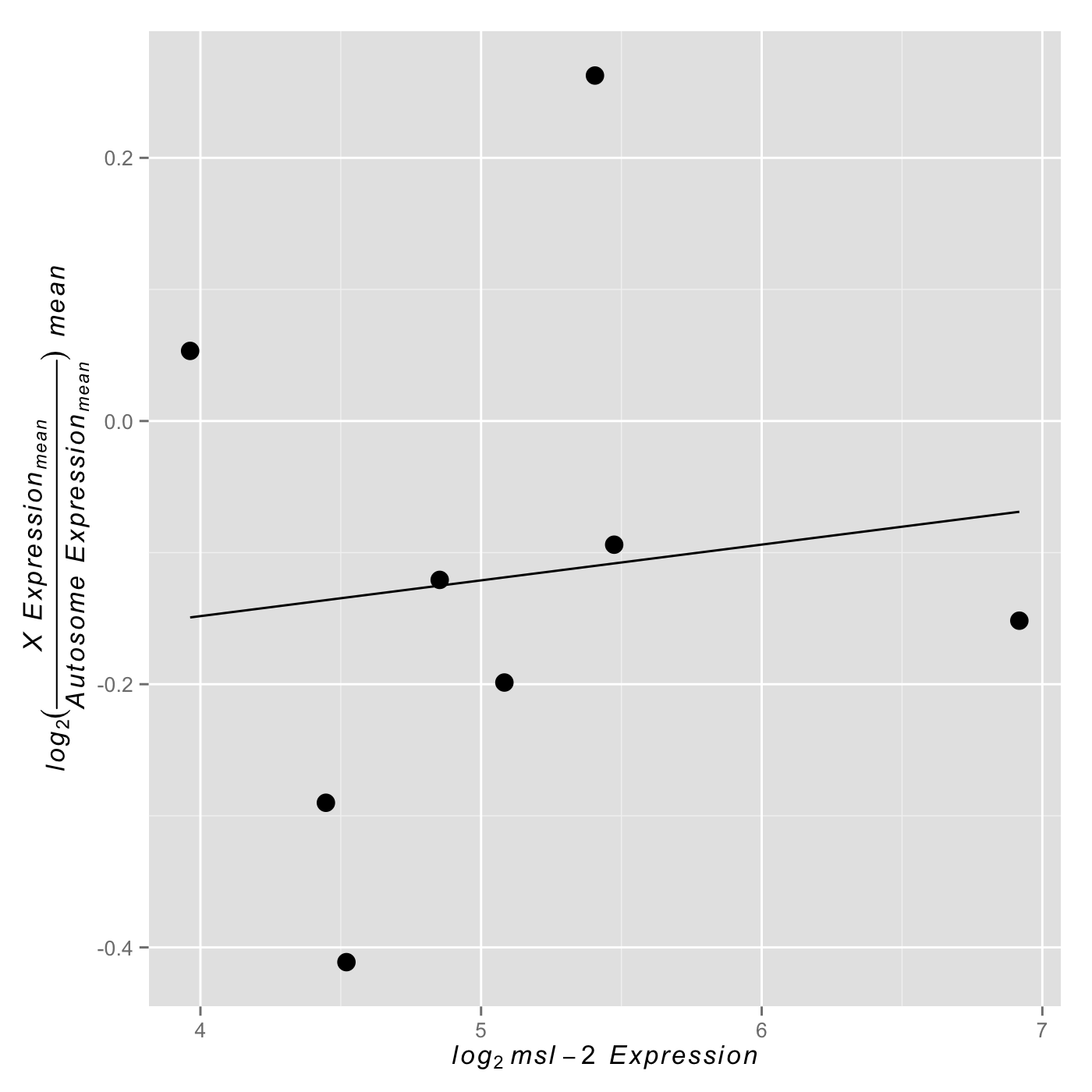

Supplement: Figure S5 — While we did not find a significant correlation, we observed a slight positive correlation between X-to-autosome expression and msl-2 among non-sex-biased genes in larval tissues (ρ ≈ 0.21, p > 0.05). [file peerj-03-771-s005.png]
